# The influence of family history of Hypertension on disease prevalence and associated metabolic risk factors among Sri Lankan adults

**DOI:** 10.1186/s12889-015-1927-7

**Published:** 2015-06-20

**Authors:** Priyanga Ranasinghe, Dilini N. Cooray, Ranil Jayawardena, Prasad Katulanda

**Affiliations:** Department of Pharmacology, Faculty of Medicine, University of Colombo, Colombo, Sri Lanka; Department of Obstetrics & Gynaecology, Faculty of Medicine, University of Colombo, Colombo, Sri Lanka; Institute of Health and Biomedical Innovation, Queensland University of Technology, Brisbane, QLD Australia; Diabetes Research Unit, Department of Clinical Medicine, Faculty of Medicine, University of Colombo, Kynsey Road, Colombo, Sri Lanka

**Keywords:** Family history, Hypertension, Prevalence, Adults, Sri Lanka

## Abstract

**Background:**

Hypertension is a major contributor to the global non-communicable disease burden. Family history is an important non-modifiable risk factor for hypertension. The present study aims to describe the influence of family history (FH) on hypertension prevalence and associated metabolic risk factors in a large cohort of South Asian adults, from a nationally representative sample from Sri Lanka.

**Methods:**

A cross-sectional survey among 5,000 Sri Lankan adults, evaluating FH at the levels of parents, grandparents, siblings and children. A binary logistic regression analysis was performed in all patients with ‘presence of hypertension’ as dichotomous dependent variable and using family history in parents, grandparents, siblings and children as binary independent variables. The adjusted odds ratio controlling for confounders (age, gender, body mass index, diabetes, hyperlipidemia and physical activity) are presented below.

**Results:**

In all adults the prevalence of hypertension was significantly higher in patients with a FH (29.3 %, n = 572/1951) than those without (24.4 %, n = 616/2530) (p < 0.001). Presence of a FH significantly increased the risk of hypertension (OR:1.29; 95 % CI:1.13-1.47), obesity (OR:1.36; 95 % CI: 1.27–1.45), central obesity (OR:1.30; 95 % CI 1.22–1.40) and metabolic syndrome (OR:1.19; 95 % CI: 1.08–1.30). In all adults presence of family history in parents (OR:1.28; 95 % CI: 1.12–1.48), grandparents (OR:1.34; 95 % CI: 1.20–1.50) and siblings (OR:1.27; 95 % CI: 1.21–1.33) all were associated with significantly increased risk of developing hypertension.

**Conclusions:**

Our results show that the prevalence of hypertension was significantly higher in those with a FH of hypertension. FH of hypertension was also associated with the prevalence of obesity, central obesity and metabolic syndrome. Individuals with a FH of hypertension form an easily identifiable group who may benefit from targeted interventions.

**Electronic supplementary material:**

The online version of this article (doi:10.1186/s12889-015-1927-7) contains supplementary material, which is available to authorized users.

## Background

Hypertension is a major contributor to the global non-communicable disease burden, affecting nearly one billion people worldwide [1,2]. It has become an important public health challenge in both developing and developed countries [3]. The rapid economic development, industrialisation and change in lifestyle seen in South Asia have led to an increasing prevalence of hypertension in this region [4]. Hypertension has been widely studied in many community surveys in South Asia, though nationally representative estimates are available only in a few countries [5]. It is estimated that in the year 2000, India had 41.5 million people with hypertension and the burden is projected to increase by another 5 million by the year 2025 [4]. The prevalence of hypertension in adults was estimated to be 23 % and 18 % in urban and rural areas respectively in the national health survey of Pakistan, conducted during 1990–94 [5]. One in four Sri Lankans over the age of 20 years was found to have hypertension, with a prevalence of 27.8 % in rural areas and 30.7 % in urban areas of the country, the latter being comparable with the prevalence of hypertension in developed countries [6,7].

Family history is an important non-modifiable risk factor for hypertension. The hereditary nature of hypertension is well established by numerous family studies [8], demonstrating associations of blood pressure among siblings and between parents and children [9]. About 30 % of the blood pressure variance can be attributed to genetic factors [10], and was found to vary from 25 % in pedigree studies to 65 % in twin studies [9]. Among various mechanisms proposed to explain the relation between hypertension and positive family history of hypertension, are the increased renal proximal sodium reabsorption [11], genetic traits related to high blood pressure such as high sodium-lithium counter-transport, low urinary kallikrein excretion, elevated uric acid level, high fasting plasma insulin concentrations, high-density LDL sub-fractions, fat pattern index, oxidative stress and body mass index, as well as shared environmental factors such as sodium intake and heavy metal exposure [12-15].

Several associations were found between family history and hypertension prevalence. In a nationwide screening program, positive family history was found to be associated with hypertension prevalence double the value of that found in persons with negative family history and was independent of weight [16]. The evidence emphasizes the need to explore the family history of hypertension even in a normotensive individual. There is strong evidence of early cardiac morphologic changes (greater left ventricular wall thickness and mass) and altered peripheral vascular capacity and responsivity to pressor stimuli among normotensive individuals with a positive family history [17]. Teenagers with hypertensive first-degree relatives constitute a special risk group that should be closely monitored [18]. Hypertension was more likely to have been previously diagnosed in screening programmes if family history was positive [16]. Studying family history of hypertension and other risk factors in healthy individuals provides a unique opportunity to explore factors leading to elevated blood pressure, long before a diagnosis of hypertension is made [19]. Positive family history therefore can be considered as an opportunity for involving direct family members in health education, as well as for early interventions and improved control of hypertension [20].

To our knowledge presently there are no detailed analysis of the association between family history of hypertension and the prevalence of the disease in a large cohort of ethnic South Asian adults. The present study aims to describe the influence of family history on hypertension prevalence and associated metabolic risk factors in a large cohort of South Asian adults, from a nationally representative sample from Sri Lanka.

## Methods

### Study population and sampling

The present study was a cross-sectional community based national survey conducted between August 2005 and September 2006 in seven of the nine provinces in Sri Lanka. Detailed sampling has been described elsewhere [21]. In brief, 5,000 adults (>18 years) were invited for the study. A multi-stage stratified cluster sampling technique used for recruitment. A total of 100 clusters of 50 adults each were divided amongst the seven provinces using a probability proportional to size (PPS) technique based on total provincial populations. In each province the required number of clusters were selected from a list of all ‘Village Officers Units’ in that province by using a computer-generated random number list. The voter registration lists of the selected ‘Village Officers Units’ were used to randomly select the first household in each cluster and a uniform criterion was used to select the remaining households. Ethical approval for the study was obtained from the Ethics Review Committee, Faculty of Medicine, University of Colombo, Sri Lanka.

### Data collection

An interviewer administered questionnaire was used to collect data, which included; age, gender, area of residence, ethnicity, level of education, household monthly income (LKR-Sri Lankan Rupees), presence of hypertension and diabetes, family history of hypertension, height, weight, waist circumference and hip circumference. Each study participant was asked by direct questioning whether any of his/her family members (living or not) had hypertension, diagnosed by a physician. The family history was evaluated at three levels, a) parents, b) grandparents and c) siblings. The options provided were ‘yes’, ‘no’, and ‘don’t know’. For the purpose of analysis those who said that they were unaware (‘don’t know’) about the family history were excluded. In addition family history was grouped into the following generations; 1st generation-siblings, 2nd generation-parents and 3rd generation-grandparents. We also looked for the presence of hypertension amongst the children of the study participants.

Data on self-reported physical activity were collected using the short version of the International Physical Activity Questionnaire (IPAQ). Height was measured using Harpenden stadiometers (Chasmors Ltd, London, UK) to the nearest 0.1 cm. Body weight was measured using a SALTER 920 digital weighing scale (SALTER Ltd, Tonbridge, UK) to the nearest 0.1 kg. Body Mass Index (BMI) was calculated as weight in kilograms divided by height squared in meters (kg/m^2^). Waist circumference (WC) was measured midway between the iliac crest and the lower rib margin at the end of normal expiration and hip circumference was measured at the widest level over the greater trochanters using a plastic flexible tape to the nearest 0.1 cm. Waist to Hip Ratio (WHR) and Waist to Height Ratio (WHtR) were calculated as waist circumference divided by hip circumference and height respectively. Seated blood pressure was measured after at least a 10-min rest with Omron IA2 digital blood pressure monitors (Omron Healthcare, Singapore). Fasting venous blood samples were obtained for glucose and lipid estimation from all participants, details of analysis have been previously described [21].

### Definitions

Hypertension was defined as systolic blood pressure > 140 mmHg and/or diastolic blood pressure > 90 mmHg and/or being on anti-hypertensive treatment [22]. Participants were considered to have diabetes if they had been previously diagnosed at a government hospital or by a registered medical practitioner. New cases (‘undiagnosed diabetes’) were diagnosed according to the American Diabetes Association (ADA) and World Health Organization (WHO) criteria [23,24]. Central obesity was classified as WC > 90 cm for males and >80 cm for females (Asian cut-offs) [25]. Obesity was defined as a BMI ≥ 23 kg/m2, based on criteria for Asians [25]. Metabolic Syndrome was defined according to the International Diabetes Federation (IDF) criteria [26]. Urban and rural sectors were defined according to the classification of the Sri Lankan government. Physical activity was classified in to three categories (‘Inactive’, ‘Moderately active’ and ‘Highly active’) based on the total MET minutes/week derived from the IPAQ short version [21].

### Statistical analysis

Data were analysed using SPSS v20 (SPSS Inc., Chicago, IL, USA) statistical software package. The significance of the differences between proportions and means was tested using z-test and Student’s t-test or ANOVA respectively. A binary logistic regression analysis was performed in all patients with ‘presence of hypertension’ as the dichotomous dependent variable (0 = hypertension absent; 1 = hypertension present) and using family history in parents, grandparents, siblings and children as the binary independent variables (0 = No, 1 = Yes). The explanatory variables selected above were subsequently included in a binary logistic regression model, a backward elimination procedure was used and a p-value of 0.10 was considered as the cut off for removal of variables. A similar binary logistic regression analysis with above dependant and independent variables was also performed separately for both males and females. We present the results of the logistic regression analysis as odds ratio controlling for confounders (age, gender, body mass index, diabetes, hyperlipidaemia and physical activity). In all statistical analyses p < 0.05 was considered significant.

## Results

### Sample characteristics

Out of the 5000 invited individuals 4482 participated in the study (89.6 % response rate). Mean age (±SD) was 46.1 ± 15.1 years and 39.5 % (n = 1772) were males. Majority were residing in rural areas (n = 3530, 78.7 %) and Sinhalese in ethnicity (n = 3877, 86.4 %). The crude prevalence of hypertension was 26.5 % (n = 1189, 95 % CI: 25.2 - 27.8), of which 579 (12.9 %) were patients with previously diagnosed hypertension. Urban adults (26.5 %) had a significantly higher prevalence of hypertension than their rural counterparts (22.9 %) (p < 0.05). Prevalence of diabetes was 12.0 % (n = 536, 95 % CI: 11.0-12.9), and 26.6 % (n = 1193, 95 % CI: 25.3-27.9) had metabolic syndrome.

### Family history and prevalence of hypertension and metabolic risk factors

The overall prevalence of family history in the population was 43.5 % (n = 1951, 95 % CI: 42.0-45.0), irrespective of disease status. A family history of hypertension (parents, grandparents or siblings) was present in 48.0 % (n = 1188, 95 % CI: 45.0-51.0) of patients with hypertension and 41.9 % (n = 3293, 95 % CI: 40.0-44.0) of participants without hypertension. In all adults the prevalence of hypertension was significantly higher in patients with a family history (29.3 %, 95 % CI: 27.3-.31.3), than those without a family history (24.4 %, 95 % CI: 22.7-26.0) (p < 0.001). A similar result was observed in females (52.2 % Vs 47.8 %), but not for male participants (41.7 % Vs 58.3 %). Presence of a family history significantly increased the risk of hypertension (OR: 1.29; 95 % CI: 1.13-1.47).

The prevalence of hypertension was higher in those with a family history of hypertension at all levels (parents, grandparents, siblings and children) than those without a family history (Table 1). Among the different ethnicities, the presence of a family history was highest in Muslims (63.4 %) followed by Burger (57.1 %) and Sinhalese (43.2 %). The prevalence of hypertension in those with a family history was significantly higher in each generations than in those without a family history (1st generation [siblings] 45.4 % Vs 22.9 %, 2nd generation [parents] 27.5 % Vs 24.0 %, p < 0.001), but not in 3rd generation [grandparents] (13.6 % Vs 22.6 %). In addition the prevalence of hypertension also increased with the number of affected generations (one −25.7 %, two-38.4 %, three-52.6 %, p < 0.001).

**Table 1:** Presence of family history in different generations in patients with and without hypertension

	Patients with hypertension (n = 1188)			p*	Patients without hypertension (n = 3293)			p**
	Family history (prevalence of hypertension)				Family history (% without hypertension)			
	Present	Absent	Not known		Present	Absent	Not known	
Parents	416 (35.0)	676 (56.9)	96 (8.1)	<0.001	1097 (33.3)	2139 (65.0)	57 (1.7)	<0.001
Grandparents	32 (2.7)	675 (56.8)	481 (40.5)	<0.001	204 (6.2)	2308 (70.1)	781 (23.7)	<0.001
Siblings	264 (22.2)	851 (71.6)	50 (4.2)	<0.001	318 (9.7)	2861 (86.9)	44 (1.3)	<0.001
Children	54 (4.5)	996 (83.8)	13 (1.1)	<0.001	22 (0.7)	2669 (81.1)	14 (0.4)	<0.001

*Comparison between prevalence of hypertension in those with and without a family history; **Comparison between the percentage without hypertension in those with and without a family history

In patients without hypertension Obesity (37.0 % vs. 25.2 %; p < 0.001), Central obesity (30.3 % vs. 21.2 %; p < 0.001) and metabolic syndrome (14.4 % vs. 11.0 %; p < 0.01) were more prevalent in those with a family history of hypertension than in those without. In this group presence of family history of hypertension increased the risk of obesity (OR: 1.36; 95 % CI 1.27-1.45), central obesity (OR: 1.30; 95 % CI 1.22 - 1.40) and metabolic syndrome (OR: 1.19; 95 % CI 1.08 - 1.30). Similarly in patients with hypertension the prevalence of Obesity (60.1 % vs. 39.5 %; p < 0.01), Central obesity (53.7 % vs. 36.4 %; p < 0.001) and metabolic syndrome (47.7 % vs. 32.3 %; p < 0.01) all were significantly higher in those with a family history of hypertension than in those without. In patients with hypertension presence of family history of hypertension increased the risk of obesity (OR: 1.54; 95 % CI 1.39 - 1.71), central obesity (OR: 1.43; 95 % CI 1.27-1.61) and metabolic syndrome (OR: 1.38; 95 % CI 1.26-1.53).

### Association of age, clinical and biochemical parameters with family history

Table 2 summarizes the association between age, clinical and biochemical parameters with family history in patients with and without diabetes. In both patients with hypertension and without hypertension, those with a family history were significantly younger and had a higher mean BMI, waist circumference, hip circumference and diastolic blood pressure. However, in the subjects without hypertension, in addition to the above mentioned parameter those with a family history also had higher LDL cholesterol and triglycerides (Table 2). In patients with hypertension the BMI, waist, hip circumferences and diastolic blood pressure significantly increased when the number of generations affected by hypertension increased from 1 to 2 (Table 3). A similar trend was also observed amongst the subjects without hypertension.

**Table 2:** Association of age, clinical and biochemical parameters with family history

	Patients with hypertension (n = 1188)		p value*	Patients without hypertension (n = 3293)		*p* value*
	Family history			Family history		
	Present	Absent		Present	Absent	
	(n = 572)	(n = 616)		(n = 1379)	(n = 1914)	
	Mean (±SD)	Mean (±SD)		Mean (±SD)	Mean (±SD)	
Age (years)	52.9 (±12.6)	59.1 (±13.4)	<0.001	40.4 (±12.3)	44.0 (±14.9)	<0.001
Body Mass Index (kg/m^2^)	24.1 (±4.4)	22.1 (±4.3)	<0.001	21.9 (±4.1)	20.8 (±3.9)	<0.001
Waist circumference (cm)	84.3 (±11.6)	79.4 (±11.6)	<0.001	77.6 (±11.5)	74.3 (±10.9)	<0.001
Hip circumference (cm)	93.4 (±9.5)	88.9 (±9.2)	<0.001	89.9 (±8.7)	86.9 (±8.6)	<0.001
Waist to hip ratio	0.90 (±0.07)	0.89 (±0.07)	0.138	0.86 (±0.07)	0.85 (±0.07)	<0.001
Systolic blood pressure (mmHg)	148.8 (±18.9)	151.1 (±19.8)	0.06	119.8 (±11.4)	118.2 (±11.8)	0.180
Diastolic blood pressure (mmHg)	86.8 (±11.0)	85.3 (±11.3)	<0.05	72.5 (±8.1)	70.7 (±8.5)	<0.05
Fasting blood glucose (mg/dl)	133.1 (±67.7)	123.3 (±58.1)	<0.05	89.0 (±26.1)	88.4 (±26.5)	0.678
Total cholesterol (mg/dl)	221.1 (±43.1)	218.7 (±44.3)	0.355	204.4 (±41.0)	200.2 (±43.4)	0.108
LDL cholesterol (mg/dl)	145.3 (±38.5)	143.8 (±37.8)	0.516	134.2 (±35.1)	130.7 (±37.9)	<0.05
HDL cholesterol (mg/dl)	46.5 (±9.6)	47.0 (±10.3)	0.399	46.8 (±10.8)	46.8 (±10.8)	0.644
Triglycerides (mg/dl)	146.7 (±76.4)	138.9 (±69.5)	0.07	116.5 (±63.4)	112.4 (±62.2)	<0.05

*Patients with and without family history

**Table 3:** Association of age, clinical and biochemical parameters with number of generations with family history

	Patients with hypertension & a family history of hypertension (n = 508)			Patients without hypertension, with a family history of hypertension (n = 1325)		
	One Generation (n = 353)	Two Generations (n = 145)	Three Generations (n = 10)	One Generation (n = 1085)	Two Generations (n = 282)	Three Generations (n = 9)
	Mean (±SD)	Mean (±SD)	Mean (±SD)	Mean (±SD)	Mean (±SD)	Mean (±SD)
Age (years)	51.8 (±12.7)	50.5 (±9.3)	45.8 (±10.3)	40.0 (±12.0)	39.2 (±11.3)	44.8 (±12.5)
Body Mass Index (kg/m^2^)	23.8 (±4.3)^#^	25.6 (±4.3)^#^	24.3 (±2.7)	21.8 (±4.0)^¥δ^	22.6 (±4.2)^¥^	24.9 (±6.9)^δ^
Waist circumference (cm)	84.2 (±10.8)^#^	88.0 (±11.8)^#^	84.2 (±7.7)	77.1 (±11.4)^¥δ^	79.2 (±10.9)^¥^	86.0 (±20.5)^δ^
Hip circumference (cm)	92.7 (±9.1)^#^	96.1 (±9.8)^#^	93.6 (±5.4)	89.5 (±8.5)^¥^	91.6 (±9.2)^¥^	94.5 (±14.2)
Waist to hip ratio	0.91 (±0.07)	0.93 (±0.07)	0.93 (±0.08)	0.86 (±0.07)	0.86 (±0.07)	0.90 (±0.07)
Systolic blood pressure (mmHg)	147.6 (±16.9)^#^	150.2 (±20.5)^*^	134.5 (±10.5)^#*^	119.6 (±11.4)	120.6 (±11.1)	125.1 (±10.6)
Diastolic blood pressure (mmHg)	86.5 (±10.6)^#^	89.6 (±11.0)^#^	84.1 (±7.9)	72.4 (±8.2)	73.0 (±8.0)	76.1 (±5.8)
Fasting blood glucose (mg/dl)	99.2 (±34.5)	105.7 (±38.5)	75.3 (±13.6)	88.4 (±23.3)	91.4 (±36.9)	82.5 (±12.9)
Total cholesterol (mg/dl)	220.5 (±43.3)	224.9 (±41.9)	204.1 (±41.8)	204.0 (±41.1)	206.0 (±40.7)	206.2 (±35.4)
LDL cholesterol (mg/dl)	144.6 (±39.4)	148.4 (±36.0)	136.8 (±41.0)	134.1 (±35.1)	134.9 (±35.7)	131.3 (±24.3)
HDL cholesterol (mg/dl)	46.2 (±9.7)^#^	46.8 (±9.7)^*^	39.5 (±5.5)^#*^	46.6 (±10.7)	47.7 (±11.0)	52.1 (±14.5)
Triglycerides (mg/dl)	148.0 (±84.7)	148.4 (±63.5)	138.5 (±59.7)	115.9 (±61.6)	115.8 (±68.1)	114.1 (±68.6)

^*#¥δ^Mean values in the same row with the same superscript are significantly different from each other (*p* < 0.05)

**Fig. 1:**
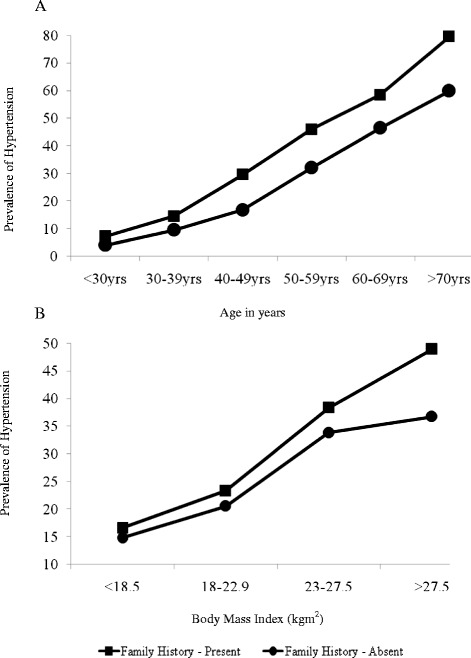
Prevalence of hypertension by, **a**) age category and **b**) BMI in patients with and without a family history of hypertension

Given the strong influences of age and BMI on the prevalence of hypertension, we tested separately the influence of family history on the prevalence of hypertension across commonly used age and BMI categories (Fig. 1). In virtually every stratum of age (Fig. 1a) and BMI (Fig. 1b), there was a clear stratification of risk for diabetes according to family history. In patients with hypertension, those with a family history had a lower total weekly MET minutes (3455) than those without a family history (3773) (p-0.130). A similar result was observed in the subjects without hypertension (4693 vs. 5387, p < 0.001). Patients with hypertension with a family history were more ‘inactive’ (18.4 % vs. 14.8 %, p-0.135) when self reported physical activity was classified in to the three categories based on the total MET minutes/week. This was not observed in non-hypertensive patients.

### Logistic regression analysis

The overall model was statistically significant and the Cox & Snell R-Square and Nagelkerke R Square values were 0 .041 and 0.06 respectively. The results indicate that in all adults presence of family history in parents (OR: 1.28), siblings (OR: 1.27) and grandparents (OR: 1.34) all were associated with significantly increased risk of developing hypertension (Table 4). Family history in grandparents and siblings were also associated with developing hypertension in both males and females independently (Table 4). However family history in parents was not associated with an increase in risk of hypertension in males.

**Table 4:** Binary logistic regression analysis in all adults, males and females

Co-variants (Family history)	Adjusted Odds Ratio (95 % CI)^α^		
	All adults (n = 4482)	Males (n = 1772)	Females (n = 2710)
Parents	1.28 (1.12 – 1.48)*	1.20 (0.90 – 1.51)	1.31 (1.11 – 1.51)*
Grandparents	1.34 (1.20 – 1.50)*	1.31 (1.21 – 1.53)*	1.17 (1.06 – 1.29)*
Siblings	1.27 (1.21 – 1.33)*	1.31 (1.11 – 1.56)*	1.24 (1.15 – 1.47)*
Children	0.90 (0.80 – 0.98)	0.89 (0.85 – 0.94)	0.91 (0.86 – 0.96)

*p <0.001; α – adjusted odds ratio controlling for confounders (age, gender, body mass index, diabetes, hyperlipidemia and physical activity)

## Discussion

To our knowledge this is the first comprehensive national survey evaluating the association between family history of hypertension, disease prevalence and metabolic risk factors from a nationally representative, large cohort of ethnic South Asian adults from Sri Lanka. In the present cohort those with a family history of hypertension are nearly 1.4 times more likely to develop hypertension than those without a family history. Previous studies from different countries have shown similar increased risks [10,16,27,28], where compared to people without a family history of hypertension, those who have a family history were 2–4 times more likely to develop hypertension [10,16]. However, the observed risks were much higher in industrialised populations than reported in the present cohort [10,27]. Furthermore our results clearly demonstrate that family history of hypertension has a graded association with hypertension, as the prevalence of hypertension increased with the number of generations with a family history.

The risk associated with family history in the current population appeared to be independent of other known risk factors, including age, anthropometric parameters (BMI) and lifestyle factors (physical activity). The Johns Hopkins Precursors Study has identified that hypertension in both mother and father has a strong independent association with elevated BP levels and incident hypertension over the course of adult life [29]. A nationwide study involving over half a million study population has described the association between family history and hypertension is independent of weight [16]. Furthermore, family history and hypertension showed a graded association, as the prevalence increased with the increasing number of generations affected. A similar study has shown that the strength of the association was related to the type and number of relatives involved [30].

Participants with a family history of hypertension displayed a significantly higher mean BMI, waist and hip circumference and diastolic blood pressure than those without a family history, irrespective of hypertension status, a finding which is in keeping with other similar studies [20,31]. Furthermore, the anthropometric parameters in the present cohort showed a graded increase with the increasing number of generations affected by hypertension. Family history of hypertension was also associated with the prevalence of obesity, central obesity and metabolic syndrome. A study carried out in Japan has identified that a maternal family history of hypertension was positively associated with the risk of overweight in children and the risk increased with increasing number of affected family members [32]. A similar result was observed in the present cohort, where in patients with and without hypertension the BMI, waist and hip circumferences and diastolic blood pressure, significantly increased with the increasing number of generations affected by hypertension. Therefore it is considered that for persons with positive family history, nutritional-hygienic recommendations to avoid overweight may be important in reducing the risk of becoming hypertensive [16]. Further, early hemodynamic, neuro-humoral, and metabolic alterations that are typical of hypertensive metabolic syndrome have been demonstrated in offspring with malignant hypertensive parents [33]. Previous studies have also demonstrated an association between the family history of hypertension and the presence of metabolic disorders and the clustering of these disorders in offspring of the affected patients [34]. Also in the present cohort, in the subjects without hypertension, in addition to mean BMI, waist and hip circumference and diastolic blood pressure, those with a family history also had higher LDL cholesterol and triglycerides. Studies have demonstrated that a positive family history of hypertension is associated with an initial increase in markers of inflammation and plaque instability in otherwise healthy young normotensive individuals, likely conveying a predisposition to develop early atherothrombosis [35] and the relative risk for developing coronary artery disease or cardiovascular death is increased in patients with a family history of hypertension [10]. Hence the family history could be used as a tool to detect not only those at risk of hypertension, but also a constellation of other cardiovascular risk factors, and they might form a group of individuals that will benefit the most from targeted risk factor interventions.

We also observed that irrespective of the disease status participants with a family history had a lower total weekly MET minutes and were more ‘inactive’ in self reported physical activity than those without a family history. Objectively measured physical activity is known to have a significant correlation with blood pressure in population-based samples from Nigeria, Jamaica and the US [36]. These findings further support the hypothesis that expression of complex traits like hypertension results from an interaction between shared genes, environments and behaviours [13,31]. Furthermore individuals with a family history of hypertension form an easily identifiable group who may benefit from targeted intervention to prevent the development of hypertension through increased physical activity. Patients with a family history were younger in age than patients without a family history of hypertension, a finding which is consistent with previous studies among adults [20] and were found to have an onset well before adolescence [37].

Family history is a common non-modifiable risk factor for most chronic non-communicable diseases, as it is a collective reflection of the genetic susceptibility, shared environments and behaviours [31]. Hence, identifying the family history will serve as a practical and useful approach [31] for public health and preventive medicine [31,38]. The advantages of family history as a risk assessment tool is the low cost, greater acceptability, and that it is a reflection of the shared genetic and lifestyle factors. Although family history by itself is a non-modifiable risk factor, it is useful for screening purposes [16] to identify high risk population long before a diagnosis of hypertension is made [19], and to target interventions and disease prevention [31]. Awareness of risk is a factor that promotes better and earlier health-related behaviour [39] and lifestyle modifications are of proven efficacy in primary prevention of hypertension [40]. Hence, overall there is promising potential for the use of family history as a public health tool aiding prevention of hypertension.

The strengths of the present study includes the large and nationally representative nature of the sample, random selection of participants out of a well-defined and homogenous target population, the high response rate and the detailed nature of clinical and demographic assessment using well-validated tools. The major limitation of the study was the cross-sectional nature. The usefulness of family history in risk prediction should be tested in large prospective studies. Furthermore, in developing countries like Sri Lanka, large sections of the community remain undiagnosed and therefore the accuracy of self-reported family history is a challenge. Family history was evaluated using a simple questionnaire. Previous studies have shown that compared with a detailed questionnaire, the simple enquiry can correctly identify the majority of individuals classified as having no significant family history; however it tends to miss a significant proportion of individuals with a positive family history, a limitation that applies to the present study as well [41]. Other potential limitations include the recall bias for family history, however previous similar studies have shown higher sensitivities for reporting family history of hypertension [42]. In order to minimize recall bias we have carried out a separate analysis on patients with newly diagnosed diabetes, which is presented as a supplementary file (Additional file 1). The prevalence of diabetes in newly diagnosed patients was significantly higher in those with a family history in siblings or in children (Additional file 1). However in the binary logistic analysis only a family history in siblings was shown to be significantly associated with presence of hypertension amongst newly diagnosed patients. Furthermore newly diagnosed patients with a family history were younger in age, and had a higher BMI, waist and hip circumference.

## Conclusions

FH and hypertension had an association in the Sri Lankan population, as the prevalence of hypertension was significantly higher in those with a FH of hypertension. We also observed an increase in the prevalence with the increasing number of generations affected. FH of hypertension was also associated with the prevalence of obesity, central obesity and metabolic syndrome. Individuals with a FH of hypertension form an easily identifiable group who may benefit from targeted interventions.

## Additional file

Additional file 1: Table S1. Presence of family history in different generations in newly diagnosed patients with hypertension. **Table S2.** Association of age, clinical and biochemical parameters with family history in patients with newly diagnosed hypertension. Table S3: Binary logistic regression analysis in all adults, males and females.

## References

[1] Kearney PM, Whelton M, Reynolds K, Muntner P, Whelton PK, He J (2005). Global burden of hypertension: analysis of worldwide data. Lancet.

[2] Global Health Observatory [http://www.who.int/gho/en/]

[3] Deedwania PC, Gupta R (2012). Hypertension in south Asians. S Asian J Prev Cardiol.

[4] Sharma S (2008). Hypertension and cardiovascular disease in South Asia: No end in sight. J Am Soc Hypertension : JASH.

[5] Ghaffar A, Reddy KS, Singhi M (2004). Burden of non-communicable diseases in South Asia. BMJ.

[6] Katulanda P, Ranasinghe P, Jayawardena R, Constantine GR, Rezvi Sheriff MH, Matthews DR (2014). The prevalence, predictors and associations of hypertension in Sri Lanka: a cross-sectional population based national survey. Clin Exp Hypertens.

[7] Kasturiratne A, Warnakulasuriya T, Pinidiyapathirage J, Kato N, Wickremasinghe R, Pathmeswaran A (2011). P2-130 Epidemiology of hypertension in an urban Sri Lankan population. J Epidemiol Community Health.

[8] Barlassina C, Lanzani C, Manunta P, Bianchi G (2002). Genetics of essential hypertension: from families to genes. J Am Soc Nephrol: JASN.

[9] Carretero OA, Oparil S (2000). Essential hypertension. Part I: definition and etiology. Circulation.

[10] Corvol P, Jeunemaitre X, Charru A, Soubrier F (1992). Can the genetic factors influence the treatment of systemic hypertension? The case of the renin-angiotensin-aldosterone system. Am J Cardiol.

[11] Simsolo RB, Romo MM, Rabinovich L, Bonanno M, Grunfeld B (1999). Family history of essential hypertension versus obesity as risk factors for hypertension in adolescents. Am J Hypertens.

[12] Williams RR, Hunt SC, Hopkins PN, Hasstedt SJ, Wu LL, Lalouel JM (1994). Tabulations and expectations regarding the genetics of human hypertension. Kidney Int Suppl.

[13] Siervogel RM (1983). Genetic and familial factors in essential hypertension and related traits. Am J Phys Anthropol.

[14] Feig DI, Kang DH, Johnson RJ (2008). Uric acid and cardiovascular risk. N Engl J Med.

[15] Munzel T, Gori T, Bruno RM, Taddei S (2010). Is oxidative stress a therapeutic target in cardiovascular disease?. Eur Heart J.

[16] Stamler R, Stamler J, Riedlinger WF, Algera G, Roberts RH (1979). Family (parental) history and prevalence of hypertension. Results of a nationwide screening program. JAMA.

[17] Muldoon MF, Terrell DF, Bunker CH, Manuck SB (1993). Family history studies in hypertension research. Review of the literature. Am J Hypertens.

[18] Kellogg FR, Marks A, Cohen MI (1981). Influence of familial hypertension on blood pressure during adolescence. Am J Dis Child.

[19] Goldstein I, Shapiro D, Weiss R (2008). How family history and risk factors for hypertension relate to ambulatory blood pressure in healthy adults. J Hypertens.

[20] van der Sande MA, Walraven GE, Milligan PJ, Banya WA, Ceesay SM, Nyan OA (2001). Family history: an opportunity for early interventions and improved control of hypertension, obesity and diabetes. Bull World Health Organ.

[21] Katulanda P, Constantine GR, Mahesh JG, Sheriff R, Seneviratne RD, Wijeratne S (2008). Prevalence and projections of diabetes and pre-diabetes in adults in Sri Lanka–Sri Lanka Diabetes, Cardiovascular Study (SLDCS). Diabet Med.

[22] Chobanian AV, Bakris GL, Black HR, Cushman WC, Green LA, Izzo JL (2003). The Seventh Report of the Joint National Committee on Prevention, Detection, Evaluation, and Treatment of High Blood Pressure: the JNC 7 report. JAMA.

[23] Expert Committee on the D, Classification of Diabetes M (2003). Report of the expert committee on the diagnosis and classification of diabetes mellitus. Diabetes Care.

[24] World Health Organization (1999). Part 1: Diagnosis and Classification of Diabetes Mellitus. Definition, Diagnosis and Classification of Diabetes Mellitus and its Complications.

[25] Consultation WHOE (2004). Appropriate body-mass index for Asian populations and its implications for policy and intervention strategies. Lancet.

[26] Alberti KG, Eckel RH, Grundy SM, Zimmet PZ, Cleeman JI, Donato KA (2009). Harmonizing the metabolic syndrome: a joint interim statement of the International Diabetes Federation Task Force on Epidemiology and Prevention; National Heart, Lung, and Blood Institute; American Heart Association; World Heart Federation; International Atherosclerosis Society; and International Association for the Study of Obesity. Circulation.

[27] Williams RR, Hunt SC, Hopkins PN, Wu LL, Hasstedt SJ, Berry TD (1993). Genetic basis of familial dyslipidemia and hypertension: 15-year results from Utah. Am J Hypertens.

[28] Masuo K, Mikami H, Ogihara T, Tuck ML (1998). Familial hypertension, insulin, sympathetic activity, and blood pressure elevation. Hypertension.

[29] Wang NY, Young JH, Meoni LA, Ford DE, Erlinger TP, Klag MJ (2008). Blood pressure change and risk of hypertension associated with parental hypertension: the Johns Hopkins Precursors Study. Arch Intern Med.

[30] Goldstein IB, Shapiro D, Guthrie D (2006). Ambulatory blood pressure and family history of hypertension in healthy men and women. Am J Hypertens.

[31] Khanna N, Sharma RS, Sidhu RS (2011). A study of the basic and derived anthropometric indices among the healthy adults (20–30 years of age) of amritsar city (punjab) having family history of hypertension. Int J Biol Med Res.

[32] Liu J, Sekine M, Tatsuse T, Hamanishi S, Fujimura Y, Zheng X (2014). Family history of hypertension and the risk of overweight in Japanese children: results from the Toyama Birth Cohort Study. J Epidemiol.

[33] Lopes HF, Bortolotto LA, Szlejf C, Kamitsuji CS, Krieger EM (2001). Hemodynamic and metabolic profile in offspring of malignant hypertensive parents. Hypertension.

[34] Wada K, Tamakoshi K, Yatsuya H, Otsuka R, Murata C, Zhang H (2006). Association between parental histories of hypertension, diabetes and dyslipidemia and the clustering of these disorders in offspring. Prev Med.

[35] Solini A, Santini E, Passaro A, Madec S, Ferrannini E (2009). Family history of hypertension, anthropometric parameters and markers of early atherosclerosis in young healthy individuals. J Hum Hypertens.

[36] Luke A, Kramer H, Adeyemo A, Forrester T, Wilks R, Schoeller D (2005). Relationship between blood pressure and physical activity assessed with stable isotopes. J Hum Hypertens.

[37] Munger RG, Prineas RJ, Gomez-Marin O (1988). Persistent elevation of blood pressure among children with a family history of hypertension: the Minneapolis Children’s Blood Pressure Study. J Hypertens.

[38] Yoon PW, Scheuner MT, Peterson-Oehlke KL, Gwinn M, Faucett A, Khoury MJ (2002). Can family history be used as a tool for public health and preventive medicine?. Genet Med.

[39] Zafar SN, Gowani SA, Irani FA, Ishaq M (2008). Awareness of the risk factors, presenting features and complications of hypertension amongst hypertensives and normotensives. JPMA The Journal of the Pakistan Medical Association.

[40] Whelton PK, He J, Appel LJ, Cutler JA, Havas S, Kotchen TA (2002). Primary prevention of hypertension: clinical and public health advisory from The National High Blood Pressure Education Program. JAMA : the journal of the American Medical Association.

[41] Wijdenes-Pijl M, Henneman L, Cross-Bardell L, Timmermans DRM, Qureshi N (2011). How does a simple enquiry compare to a detailed family history questionnaire to identify coronary heart disease or diabetic familial risk?. Genetics in medicine : official journal of the American College of Medical Genetics.

[42] Bochud M, Burnier M, Paccaud F, Falconnet C, Mooser V, Both N (2004). Patients’ sibling history was sensitive for hypertension and specific for diabetes. J Clin Epidemiol.

