# Supplementary material for: The influence of family history of Hypertension on disease prevalence and associated metabolic risk factors among Sri Lankan adults
Source: BMC Public Health. 2015 Jun 20;15:576. doi: 10.1186/s12889-015-1927-7 (PMC4475303; doi:10.1186/s12889-015-1927-7)
Supplement: Additional file 1: Table S1. — Presence of family history in different generations in newly diagnosed patients with hypertension. Table S2. Association of age, clinical and biochemical parameters with family history in patients with newly diagnosed hypertension. Table S3: Binary logistic regression analysis in all adults, males and females. [file 12889_2015_1927_MOESM1_ESM.doc]

**Additional file 1**

**Table S1:** Presence of family history in different generations in newly diagnosed patients with hypertension

|  | Number with Family history (hypertension prevalence) | | | p value* |
| --- | --- | --- | --- | --- |
|  | Present | Absent | Not known |  |
| Parents | 194 (15.0) | 379 (15.1) | 36 (38.7) | 0.984 |
| Grandparents | 13 (6.0) | 370 (13.8) | 226 (22.4) | 0.064 |
| Siblings | 104 (24.6) | 473 (14.2) | 20 (31.3) | <0.001 |
| Children | 20 (47.6) | 498 (15.7) | 4 (22.2) | <0.001 |

^*^ Comparison between prevalence of hypertension in those with and without a family history

**Table S2** Association of age, clinical and biochemical parameters with family history in patients with newly diagnosed hypertension

|  | Family history | | p value* |
| --- | --- | --- | --- |
|  | Present  Mean (±SD) | Absent  Mean (±SD) |  |
| Age (years) | 49.3 (±12.3) | 56.5 (±14.3) | <0.001 |
| Body Mass Index (kg/m^2^) | 24.2 (±4.6) | 22.1 (±4.4) | <0.001 |
| Waist circumference (cm) | 84.0 (±11.7) | 79.0 (±11.8) | <0.001 |
| Hip circumference (cm) | 93.2 (±9.5) | 88.9 (±9.4) | <0.001 |
| Waist to hip ratio | 0.9 (±0.08) | 0.9 (±0.08) | 0.061 |
| Systolic blood pressure (mmHg) | 149.9 (±12.2) | 152.7 (±15.6) | <0.01 |
| Diastolic blood pressure (mmHg) | 88.4 (±9.1) | 87.1 (±9.8) | 0.085 |
| Fasting blood glucose (mg/dl) | 97.7 (±33.9) | 95.8 (±29.4) | 0.454 |
| Total cholesterol (mg/dl) | 222.8 (±39.9) | 217.8 (±42.0) | 0.137 |
| LDL cholesterol (mg/dl) | 147.0 (±34.1) | 142.7 (±35.8) | 0.128 |
| HDL cholesterol (mg/dl) | 47.0 (±9.9) | 47.9 (±11.1) | 0.299 |
| Triglycerides (mg/dl) | 143.7 (±76.2) | 134.8 (±70.6) | 0.145 |

* - patients with and without family history

**Table S3:** Binary logistic regression analysis in all adults, males and females

|  | Adjusted odds ratio (95% CI)^α^ | | |
| --- | --- | --- | --- |
| Co-variants (Family history) | All adults | Male | Female |
| Parents | 1.10 (0.94 – 1.26) | 1.10 (0.99 – 1.21) | 1.26 (0.96 – 1.56) |
| Grandparents | 1.08 (0.94 – 1.22) | 1.16 (0.90 – 1.52) | 1.02 (0.86 – 1.18) |
| Siblings | 1.32 (1.11 – 1.53)^*^ | 1.22 (1.06 – 1.38)^*^ | 1.36 (1.14 – 1.58)^§^ |
| Children | 0.91 (0.88 – 0.94) | 0.90 (0.85 – 0.94) | 0.95 (0.87 – 1.08) |

* - p < 0.001, § - p <0.05; α – adjusted odds ratio controlling for confounders (age, gender, body mass index and physical activity)
